# Pressure Pain Thresholds and Central Sensitization in Relation to Psychosocial Predictors of Chronicity in Low Back Pain

**DOI:** 10.3390/diagnostics13040786

**Published:** 2023-02-19

**Authors:** Anke Steinmetz, Franziska Hacke, Karl-Stefan Delank

**Affiliations:** 1 Physical and Rehabilitation Medicine, Department of Trauma, Reconstructive Surgery and Rehabilitation Medicine, University Medicine Greifswald, 17475 Greifswald, Germany; 2 Department of Geriatrics, Martin-Luther-University Halle-Wittenberg, 06120 Halle/Saale, Germany; 3 Department of Orthopedic and Trauma Surgery, Martin-Luther-University Halle-Wittenberg, 06120 Halle/Saale, Germany

**Keywords:** pressure pain thresholds, central sensitization, Örebro Musculoskeletal Pain Screening Questionnaire (ÖMPSQ), yellow flags, cLBP, sleep disorders, Pittsburgh Sleep Quality Index (PSQI)

## Abstract

(1) Background: Peripheral, as well as central, sensitization have been described in chronic low back pain (cLBP). The purpose of this study is to investigate the influence of psychosocial factors on the development of central sensitization. (2) Methods: This prospective study investigated local and peripheral pressure pain thresholds and their dependence on psychosocial risk factors in patients with cLBP receiving inpatient multimodal pain therapy. Psychosocial factors were assessed using the Örebro Musculoskeletal Pain Screening Questionnaire (ÖMPSQ). (3) Results: A total of 90 patients were included in the study, 61 (75.4% women, 24.6% men) of whom had significant psychosocial risk factors. The control group consisted of 29 patients (62.1% women, 37.9% men). At baseline, patients with psychosocial risk factors showed significantly lower local and peripheral pressure pain thresholds, suggesting central sensitization, compared to the control group. Sleep quality, measured by the Pittsburgh Sleep Quality Index (PSQI), was also correlated with altered PPTs. After multimodal therapy, all participants reported increased local pain thresholds compared to at admission, independent of psychosocial chronification factors. (4) Conclusions: Psychosocial chronicity factors measured using the ÖMPSQ have a significant influence on pain sensitization in cLBP. A 14-day multimodal pain therapy increased local, but not peripheral, pressure pain thresholds.

## 1. Introduction

Chronic musculoskeletal pain is associated with reduced pressure pain thresholds [1,2,3,4,5,6]. In chronic pain, this phenomenon occurs not only locally, but also on peripheral locations far from the primary pain region. As such, in the context of central sensitization, pain thresholds decline at a distance from the original pain region [1,4,6]. In contrast, generalized hyperalgesia is not observed in patients with new-onset or acute pain syndromes [1,7,8].

However, it is not only chronic pain patients who experience a lowering of pain thresholds. Psychological disorders can also have an influence on pain sensitivity. Patients with major depression showed significantly reduced pressure pain thresholds compared to healthy control subjects [9,10]. With regard to therapeutic options for improving hyperalgesia, mainly individual treatment methods (e.g., training on the bicycle ergometer, segmental stabilization exercises) have been investigated for their potential in changing lowered pressure pain thresholds. Various studies have showed desensitization directly after completing a therapy session [7,11,12]. Only Cho et al. investigated an exercise program over several weeks and was also able to demonstrate an increase in pressure pain thresholds [13]. However, it is not yet known how a complex inpatient therapy program affects central and peripheral sensitization in contrast to individual outpatient therapies.

Especially in the case of degenerative spinal pathologies, conservative or combined treatment approaches with minimally invasive therapies have been discussed and investigated in recent years [14]. Precise diagnostics in chronic back pain must of course collect and evaluate clinical and radiological findings in order to be able to interpret the function and morphological changes, as well as psychosocial factors [15]. Because chronic pain, especially back pain, often occurs in combination with psychological disorders, it is frequently difficult to differentiate the cause of the hyperalgesia. Meints et al. demonstrated that pain sensitization was associated with greater catastrophizing [16]. The question arises whether chronic back pain patients with typical psychosocial factors and predictors for chronification also have lowered pressure pain thresholds compared to solely back pain patients.

Difficulties falling asleep, frequent awakenings, reduced sleep time and reduced sleep quality are factors that patients with chronic low back pain suffer from significantly more often than healthy people [17,18,19,20]. Despite the use of pain medication, the estimated prevalence of sleep disturbance is 58.7% [21]. A total of 42% of patients with chronic low back pain sleep less than six hours per night, and about one fifth of them even report less than four hours of sleep [22]. The risk of developing sleep problems increases up to 3.8 fold after a week of experiencing back pain [20]. Alsaadi et al. were able to demonstrate a bidirectional relationship between pain and sleep problems. The day after poor sleep showed increased pain intensity and, conversely, after days of severe pain, sleep quality decreased [23].

Patients affected by both back pain and sleep disturbances also frequently develop depressive episodes and anxiety disorders. Conversely, back pain patients with depressive episodes and anxiety disorders are additionally more often diagnosed with sleep disorders [24].

Furthermore, reduced sleep quality is also highly relevant in chronic pain patients. Although statistically significant correlations between chronic lumbar back pain and sleep disturbances have already been demonstrated [17,18,19,20], and chronic pain is already associated with reduced pressure pain thresholds [1,3,6], little is known about how far sleep affects pressure pain thresholds in chronic low back pain. Only for patients with rheumatoid arthritis has an association of sleep disturbances with lowered pressure pain thresholds been detected [25].

The influence of sleep on central sensitization mechanisms has gained particular attention since the recently introduced pain mechanism of nociplastic pain developed by the IASP [IASP website (https://www.iasp-pain.org/resources/terminology/?ItemNumber=1698, accessed on 15 January 2023]). Sleep disorders are listed here as one of the associated comorbidities of nociplastic pain [26].

The aim of this study was to investigate to what extent psychosocial factors, indicating a risk of developing chronic pain, influence central nociceptive sensitization processes among patients with chronic lumbar back pain. The primary question was whether patients with chronic low back pain and psychosocial risk factors exhibited generalized hyperalgesia in the context of central pain processing with reduced remote pain thresholds, in addition to a reduction in local pressure pain thresholds. Furthermore, the effect of 14-day multimodal pain therapy on local and peripheral pressure pain thresholds should be investigated, with particular focus on the potential dependence or association with the psychosocial risk factors. To our knowledge, this aspect has not yet been addressed in the literature. Secondly, the impact of sleep disturbances on peripheral and central pain sensitization in patients with cLBP should be investigated in detail to understand to what extent differences in both the quality and quantity of sleep are evident.

It is of particular interest whether inpatient multimodal pain therapy is able to eliminate local and central sensitization mechanisms and lead to general desensitization.

## 2. Materials and Methods

### 2.1. Study Design

A cross-sectional design with a prospective follow-up was used to investigate local and peripheral pressure pain thresholds. Patients with psychosocial risk factors (case group) were compared with patients without psychosocial risk factors (control group). The case group consisted of patients with chronic lumbar pain syndromes and a high risk of chronicity, defined by the cut-off of 100 points (or more) in the Örebro Musculoskeletal Pain Screening Questionnaire (ÖMPSQ) [27]. The control group consisted of patients with chronic lumbar pain syndromes and an ÖMPSQ score lower than 100, with a therefore assumed lower risk of chronicity [27].

The investigator of the pressure pain thresholds was blinded to group membership to avoid measurement bias. The research project was reviewed and approved by the Ethics Committee of the Medical Faculty of the University (ethics committee no. 2014-81), in accordance with the declaration of Helsinki of 1975 (in the current, revised version). Written informed consent was obtained from all patients. The study was retrospectively registered at the German Register of Clinical Trials (DRKS00028286).

### 2.2. Participants

Participants were recruited from patients treated in a conservative orthopedic ward at a University Hospital. Patients suitable for the study were comprehensively informed about the procedure and objectives of the study on the day of admission and included in the study after giving their written consent. A total of 114 patients were recruited from 21 July 2014 to 29 February 2016. Inclusion criteria included the presence of chronic lumbar back pain for at least a twelve-weeks duration, aged over 18 years, and sufficient knowledge of the German language. Exclusion criteria were previous spinal surgery, known traumas, infections or tumors of the spine. Female patients were not allowed to be pregnant or breastfeeding.

The case number calculation (assuming a mean effect size of d = 0.5, as well as α = 0.05 and β = 0.80) indicated a group size of 41 patients each in order to obtain sufficient power. This number of cases could not be achieved in the control group despite several extensions of the implementation period, as the patient collective mainly comprised patients with a high risk of chronicity. This resulted in an unevenly distributed sample size of 61 case patients and 29 control patients.

### 2.3. Pressure Pain Thresholds

Pressure pain thresholds were determined using the FORCE ONE FDIX DIGITAL FORCE GAGE digital pressure algometer from Wagner Instruments (Riverside, CT, USA). The algometer was set to measure at a rate of 100 readings per second and always displayed the highest pressure measured. The round attachment surface had a diameter of 1.12 cm. Newton (N) was set as the unit of measurement on the device. Later, it was converted to kilopascals (kPa) to enable comparability with other studies.

On the day of admission, the patients were comprehensively informed about the study. Subsequently, after consenting to participate, the first measurement of the pressure pain thresholds took place (T0). The pain thresholds were determined locally over the lumbar spine and peripherally on the extremities. Local measurements were carried out in the least painful resting position for the patient, for example, in the standing or prone position in the area of the facet joints of the fourth lumbar vertebra. The pressure pain thresholds of the extremities were determined in the sitting position. For the lower extremity, measurements were taken centrally over the tibialis anterior muscle and for the upper extremity, measurements were taken over the deltoid muscle about 5 cm caudal to the acromion.

All measurements were performed bilaterally and repeated three times, and they were not measured again at exactly the same point to avoid influencing the pain threshold by the previous measurements. The time interval between measurements over the same muscle was approximately 10 s [7,28]. After 14 days of multimodal therapy, mechanical pain thresholds were measured again on the eve of discharge (T1) to investigate the influence of inpatient pain therapy on pressure pain thresholds.

### 2.4. Örebro Musculoskeletal Pain Screening Questionnaire (ÖMPSQ)

The ÖMPSQ was developed to determine the individual risk of developing chronic/persistent musculoskeletal pain and contains questions addressing psychosocial factors, which have also been described as “yellow flags”, contributing to the chronification process.

In the present study, this instrument was used to assess the presence of “yellow flags” in patients with chronic back pain. The cut-off set by Linton at 90 and 105 points (from 212 points maximum) is now outdated in the literature. According to a study by Linton and Boersma, with a score of 100 points, the specificity (74%) roughly corresponds to the sensitivity (76%), which is why this value was chosen as the cut-off here [27]. With a score of at least 100, increased psychosocial risk factors contributing to the persistence of back pain problem was assumed.

### 2.5. German Pain Questionnaire

For this study, the following parameters of the German Pain Questionnaire (2006) were used and evaluated: von Korff Questionnaire (pain intensity, disability points, severity); Hospital Anxiety and Depression Scale (HADS); and general well-being.

The combination of pain intensity, disability points and severity, according to von Korff, describe the grade of pain severity (Grade I–IV).

Severity according to von Korff was described as follows: 0 = no pain; 1 = low pain intensity (<50) and low pain-related impairment (<3 disability points); 2 = high pain intensity (>50), low pain-related impairment (<3 disability points); 3 = high pain-related impairment, moderately limiting (3–4 disability points); and 4 = high pain-related impairment, severely limiting (5–6 disability points).

The Hospital Anxiety and Depression Scale (HADS) is a screening questionnaire for anxiety and depression. For both parameters, a value greater than seven is considered borderline and a value greater than ten is considered abnormal [29].

General well-being was determined using the Marburg Questionnaire on Habitual Well-being (MFHW). A score of less than ten points indicates low general well-being.

### 2.6. Pittsburgh Sleep Quality Index (PSQI)

The German version of the PSQI was used to assess the subjective sleep quality of the last four weeks before admission. The total score can assume values between 0 and 21. If the cut-off of five points was exceeded, a patient was defined as a poor sleeper [30]. In addition, the average sleep time (PSQI: question 4) was considered separately. The six-hour mark was chosen as the cut-off (analogous to Marty et al., Artner et al. [17,22]).

### 2.7. Multimodal Pain Therapy

During the inpatient stay, the patients received multimodal pain therapy. The therapy consisted of an individual combination of the following therapy methods: interventional pain therapy; optimization of oral pain medication; manual medicine; physiotherapy; sensomotoric training; medical training therapy; relaxation methods; psychoeducational training; and psychotherapy. As a rule, about 30 passive and active therapy sessions of 30 min each on average took place within 14 days.

### 2.8. Statistics

For the study design, a sample size calculation was carried out using the program nQuery. The data obtained were transferred to Excel (Microsoft) and collected. IBM SPSS Statistics 22.0 for Windows was then used for the statistical evaluation and the creation of tables and graphics. The statistical processing of the metric variables was initially carried out using descriptive statistics. Means, standard deviations and 95% confidence intervals were calculated and presented. The groups were tested for homogeneity with regard to gender, age and BMI using the Chi² test and the t-test. Furthermore, mean differences of the pressure pain thresholds were analyzed with the help of two-sided t-tests for paired and unpaired samples. Missing values were excluded list by list. The test for equality of variance of the groups presented was carried out in each case using Levene’s test. In order to determine the pairwise correlation of the individual test characteristics with each other, bivariate correlations were carried out using Pearson’s correlation coefficient. The significance level was set at *p* < 0.05 in the study design.

## 3. Results

### 3.1. Demographics

Of the 90 patients included, 61 patients had an elevated ÖMPSQ score of > 100 points and were included in the case group. The control group (ÖMPSQ score ≤ 100 points) comprised a total of 29 patients. The age of all included patients ranged between 26 and 90 years. The body mass index showed values above normal weight on average for both patient groups. A total of 41 of the patients examined had a BMI above 30 kg/m² and thus manifested obesity. Neither group showed significant differences of gender distribution, mean age or BMI (see Table 1).

### 3.2. Questionnaires

The case and control groups showed significant differences (*p* < 0.05) in the questionnaire scores regarding pain intensity, severity of chronification according to v. Korff, depression, anxiety and well-being (see Table 2).

With regard to the factors of anxiety and depressiveness, the mean values of the case group with 9.38 and 9.03 points, respectively, were in the borderline range of abnormality. Correspondingly, 41% and 36% of the patients in the case group showed abnormal values of over 10 points. In the control group, the average values of 6.66 and 6.38 were within the normal range. Only 17.2% and 13.8% of the patients in the control group had abnormal scores. While both patient groups were, on average, assigned a high-pain-related impairment of a moderately limiting character according to von Korff (severity level 3 according to von Korff), the subjective pain intensity with 72.23 (of 100) points was on average significantly higher in the case group compared to the control group (60.40 points) (*p* = 0.003).

Sleep quality was reduced on average in both groups. A total of 67 out of the 90 patients were found to be poor sleepers. The prevalence of sleep disorders in this cohort was thus 74.4%. In the case group, the percentage was even higher at 80.3% compared to the control group (62.1%). The PSQI score achieved, with an average of 10.38 points, was also significantly different from the control group’s score of 7.64 points.

The general well-being was only limited in the case group. With an average of 7.86 points, the score fell below the ten-point limit, while patients in the control group were still within the normal range with an average of 13.52 points (see Table 2).

### 3.3. Pressure Pain Thresholds

Pressure pain thresholds of the back measured at T0 were significantly lower in the case group compared to the control group (*p* = 0.047). In this group, decreased PPTs were not only displayed over the facet joints, but also peripherally over the deltoid (*p* = 0.044) and tibialis ant. (*p* = 0.046) muscles, indicating central sensitization in the patients with psychosocial factors (see Table 3). In the control group, PPTs of the back and the deltoid muscle were nearly identical, while even higher pressures were allowed above the tibialis anterior muscle. Overall, higher pressures were tolerated over the tibialis anterior muscle than over the deltoid muscle. The pain thresholds measured locally on the back were lower than the peripheral thresholds in both groups (see Table 3).

The inpatient treatment of the investigated back pain patients with multimodal pain therapy resulted in a significant improvement in the mechanical pain threshold above the spine both in the case group and in the control group (without increased psychosocial factors) at T1 (Table 4). In the case group, PPTs continued to be lowered peripherally and, in fact, were significantly reduced further, so that therapy did not lead to a decline in central sensitization. In the control group, peripheral PPTs showed no significant changes compared with T0.

### 3.4. Correlations of Demographic Factors, Questionnaire Scores and PPTs

Finally, bivariate correlations between the PPTs, questionnaire scores and demographic variables were considered (see Table 5). This showed that with higher age, less anxiety (*p* = 0.015) was reported. The local pressure pain thresholds on the back were also less sensitive (*p* = 0.001). Furthermore, gender also had an influence on pain sensitivity. Men had higher local (*p* = 0.015) and peripheral pressure pain thresholds than women (*p* = 0.001). The higher the score in the ÖMSPQ, the higher the individual scores for depression (*p* < 0.001), anxiety (*p* = 0.001), pain intensity (*p* < 0.001) and degree of impairment according to von Korff (*p* < 0.001). General well-being, on the other hand, decreased with an increase in ÖMPSQ score (*p* < 0.001). PSQI scores also correlated positively with scores for depression (*p* = 0.007), anxiety (*p* < 0.001) and pain intensity (*p* = 0.021). General well-being was again lower the higher the PSQI score increased (*p* = 0.033).

## 4. Discussion

At baseline (T0), patients in the case group showed significantly lower pressure pain thresholds both locally and peripherally. As such, central sensitization can be assumed compared to the control group. This suggests that psychosocial chronicity factors, assessed using the ÖMPSQ at T0, are associated with pain sensitization in chronic lumbar back pain. The more pronounced the “yellow flags” are, the lower the pressure pain thresholds become.

In the literature, the overall ÖMPSQ score has not yet been linked to pressure pain thresholds. However, evidence can be found for the association of individual psychological chronicity factors, such as depression or anxiety, with pressure pain thresholds. A higher questionnaire score in anxiety and/or depression is associated with lower pressure pain thresholds [2,5,9,10]. Interestingly, Lautenbacher et al. postulated completely opposite results. Patients with depression had significantly higher pressure pain thresholds [31]. Regarding the risk factors of pain intensity and severity of impairment, a review by Hübscher et al. can be considered, in which the level of pressure pain thresholds made it possible to distinguish between patients with and without pain, without being able to predict pain intensity or severity of impairment [32]. The present results only allow a group classification (high vs. low psychosocial factors), and not a direct correlation of pain thresholds to questionnaire scores. It is therefore neither possible to predict the exact risk of chronicity via pain thresholds nor to determine the pain thresholds via the questpptionnaire scores.

One of the main results was that pain sensitization does not only occur at the site of the pain focus, but that it rather leads to generalized hyperalgesia. Comparable results were also found in other studies. Patients with chronic back pain had significantly lower pressure pain thresholds (ppts) both locally and peripherally than healthy controls [1,4,6]. In addition, Meints et al. showed that regardless of increased pain sensitivity compared to healthy controls, within the cLBP group, greater catastrophizing was associated with both greater experimental pain sensitivity and clinical pain, with deep-tissue hyperalgesia mediating between the extent of catastrophizing and clinical pain [16]. That psychosocial factors, such as catastrophizing, contribute to central pain sensitization has been discussed in several studies [33,34], but to our knowledge no studies have been conducted using a psychosocial predictive score for the development of chronic pain syndrome, such as the ÖMPSQ, so far. Based on this, the present study underscores that cLBP patients with varying degrees of psychosocial factors differ with respect to the fact that central sensitization takes place. These findings are in line with recent findings by Aoyagi et al., showing that the 2011 FM survey (Fibromyalgia Criteria and Severity Scales) identifies a subgroup of cLBP patients who exhibit central sensitization in association with greater catastrophizing, anxiety and depressive symptoms [35].

When comparing the absolute measured values in the literature, large differences can be found. While the mean values of this study range between 222 kPa and 318 kPa, depending on the case or control group and pressure location, O‘Neill et al. measured significantly higher values with an average of 450 kPa or even 680 kPa in patients with chronic lumbar back pain [1,28]. Starkweather et al., on the other hand, described rather lower values with an average of 180 kPa [8]. These discrepancies can be explained, for example, by the use of different pressure algometers with headpieces of different sizes. Furthermore, the composition of the patient collective could also explain the poor comparability. This study included patients who required 14 days of inpatient pain therapy, which implies high suffering levels and a long course of the disease.

A study by Corrêa et al., which also included healthy subjects, however, shows a similar pain threshold level in the results [6]. Unfortunately, the study does not provide any information on the size of the algometer headpiece used, so it is not possible to determine beyond doubt whether the results are really comparable. Nevertheless, this study provides the opportunity to compare the pain thresholds of the control group of the present study with the pain thresholds of completely healthy subjects. Patients with chronic lumbar back pain showed lumbar ppts of 253 kPa and ppts of 262 kPa over the tibialis anterior muscle [6], which roughly correspond to the pressure pain thresholds of the present study. Healthy subjects without back pain showed lumbar ppts of 343 kPa, which were significantly higher than those of the back pain patients in this study (222 kPa and 277 kPa). This suggests that in the present study, local sensitization took place in both the case and control groups, which was to be expected due to the chronification of the pain syndrome that had taken place. However, the mean values of the peripheral pain thresholds in the healthy subjects (322 kPa) of Corrêa hardly exceed our values of the patients with back pain without psychosocial risk factors (319 kPa) [6]. This supports our hypothesis that no central sensitization took place in the control group of our study.

After 14 days of multimodal inpatient treatment, all participants showed higher local pain thresholds than at admission (T0). This phenomenon was present in the case and control groups, independent of psychosocial chronification factors. Consequently, the therapy must have contributed to the desensitization of the previously hyperalgesic areas. An increase in pressure pain thresholds after various physical therapy procedures has been described in the literature [7,11,12]. In the present study, infiltration techniques, oral analgesics and psychological therapies may additionally have supported desensitization. There are very few studies on these widespread therapeutic approaches, especially on drug effects, in the context of pressure pain thresholds. For benzodiazepines, Vuilleumer et al. could not prove an antihyperalgesic effect. Peripherally, however, inpatient treatment led to further sensitization instead of a subsequent reduction in central sensitization [36]. Nevertheless, Vaegter et al. also demonstrated improvements in local ppts on the back and no changes in remote trapezius ppts during 12-week cognitive functional therapy (a physiotherapy-guided intervention with physical, lifestyle and psychological targets), indicating no change in central sensitization [37].

From the present results, it can be concluded that the local changes regarding sensitization are reduced by 14-day pain therapy. The central processes, represented by peripheral pain thresholds, however, do not seem to have decreased in this short period of time. On the contrary, peripheral sensitization even seemed to continue in the case group. Provided that pain threshold measurement is subject to good test–retest reliability [38,39], it remains to be considered which factors could be responsible for the decrease in peripheral pain thresholds. It might well be that proinflammatory mechanisms continue to be present peripherally and maintain sensitization, which has been resolved locally by the treatment. Perhaps the local pain relief also causes a kind of shift in perception. The back is no longer the center of attention, so peripheral pain stimuli are not superimposed and rather reach the consciousness. Further studies are obviously necessary in this regard.

The prevalence of sleep disturbances was 74.4% in the entire patient population. This value is consistent with the data from the literature for patients with chronic lumbar back pain [19,20,22]. The prevalence of sleep disturbance was even higher with an ÖMPSQ score above 100 (80,3%). Furthermore, in accordance with the literature, a positive correlation of the PSQI to the individual chronicity factors mapped by the HADS-D, HADS-A, pain intensity and negatively to the MFHW can be observed [24]. It can therefore be stated that chronic back pain patients with psychosocial chronicity factors sleep less well or that pain patients who sleep less well tend to have psychosocial factors. The exact relationship between the factors cannot be deduced from the present results.

Furthermore, this study showed that poor sleepers with a PSQI score of more than five points have significantly lower pressure pain thresholds over the facet joints and the deltoid muscle. How sleep quality affects the pressure pain thresholds of chronic back pain patients has not yet been discussed in studies, so no comparative values are available. Because the PSQI correlates with other risk factors, it is also possible that only these are represented here.

Another factor that supports the importance of sleep in this context is absolute sleep time. Subjectively, this was around six hours on average. In the literature, subjective sleep times range between six and eight hours on average, regardless of the presence of chronic back pain [18,19,23,40]. Artner et al. show in their study that 42% of back pain patients sleep less than six hours [22]. In the present patient collective, about 45% of the subjects slept less than six hours per night. Additionally, they showed significantly reduced pain thresholds in comparison. This phenomenon was observed both locally and peripherally. Patients who sleep less than six hours seem to be prone to generalized hyperalgesia. Age may have acted as a possible confounding factor in this evaluation. The patients with less sleep time were significantly younger. However, a study by Donat et al. shows that pressure pain thresholds do not differ significantly in different age groups [41]. At most, there is a slight decrease in values with an increase in age, but not an increase. The correlations drawn in this study also do not reveal any correlations between age and peripheral pressure pain threshold. Nevertheless, there is a lack of comparable study results that support the influence of sleep time on pressure pain thresholds. It becomes clear that greater attention should be paid to the topic of sleep in research and therapy as a whole.

### Limitations

Over the observation period, a total of 114 patients were recruited and of these, 90 patients were finally included in the study. A total of 61 patients were assigned to the case group and 29 patients were assigned to the control group. The recruitment phase was extended from the originally planned eight months to nineteen months. In total, even more than the originally planned 82 participants were recruited, but there was still asymmetry with regard to group size, which could have reduced the planned power. The patient collective of the conservative orthopedic ward of a University Hospital mainly comprised patients with a high risk of chronification. The sample size is comparable to that of similarly structured studies on the topic of pressure pain thresholds [7,8,10,25]. Asymmetries in group size can also be observed here.

Above all, the exclusion of all patients who had undergone back surgery further reduced the number of patients who could be recruited. On the other hand, studies have not yet investigated the extent to which spinal surgery influences pressure pain thresholds. As such, distortion by this could not be ruled out. Another minimizing factor was the fact that a control examination was to be carried out in all patients after the 14-day pain therapy. Because 22 patients who were already recruited discontinued the treatment prematurely, there was a further reduction in the number of patients.

Another possible limiting factor to be discussed would be that patients with a pathological score in the HADS were not excluded. However, as no correlations between the HADS-A and HADS-D with pressure pain thresholds could be shown, it can be assumed that abnormal values in the HADS did not distort the results. Nevertheless, in future studies, the presence of depression or anxiety should definitely be excluded.

## 5. Conclusions

In this study, we were able to demonstrate for the first time that central sensitization in patients with chronic back pain is associated with the extent of psychosocial factors known as “yellow flags” measured with the ÖMPSQ, although we cannot conclude the exact relationship from the results. Furthermore, a 14-day multimodal pain therapy influences local pressure pain thresholds, which are significantly reduced in both (case and control) groups. Central sensitization, however, could not be influenced by the therapy; unexpectedly, it even increased. Further studies are warranted to understand these mechanisms and to develop therapy strategies for the reduction and reversal of central sensitization.

## Figures and Tables

**Table 1:** Demographics.

ÖMPSQ	>100 pts. (Case Group)	<100 pts. (Control Group)	*p*
N	61	29	N.A.
Sex Female (N/%) Male (N/%)	46 (75)	18 (62)	0.192
	15 (25)	11 (38)	
Age (yrs) Mean (SD)	65.1 (12.5)	63.8 (11.8)	0.638
BMI (kg/m^2^) Mean (SD)	30 (5.5)	29.5 (5.3)	0.715

N: number; ÖMPSQ: Örebro Musculoskeletal Pain Screening Questionnaire.

**Table 2:** Questionnaire scores in case and control group.

		N	M	SD	SEM	*p*
ÖMPSQ	Case	61	133.28	22.496	2.880	<0.001
	Control	29	87.59	16.240	3.016	
HADS-A	Case	61	9.03	4.626	0.592	0.009
	Control	29	6.38	3.802	0.706	
HADS-D	Case	61	9.38	4.140	0.530	0.004
	Control	29	6.66	3.810	0.708	
Pain intensity	Case	61	72.23	15.959	2.043	0.003
	Control	29	60.40	19.985	3.711	
Pain severity v. Korff	Case	61	3.75	0.977	0.125	0.009
	Control	29	3.03	1.239	0.230	
MFHW	Case	58	7.86	8.008	1.051	0.003
	Control	29	13.52	8.175	1.518	
PSQI	Case	60	10.38	4.748	0.613	0.009
	Control	28	7.64	3.783	0.715	
Sleeping time [h]	Case	60	5.80	1.619	0.209	0.206
	Control	28	6.30	1.940	0.367	

N: number; M: mean; SD: standard deviation; SEM: standard error of the mean; P: *p*-value; ÖMPSQ: Örebro Musculoskeletal Pain Screening Questionnaire; HADS-A: Hospital Anxiety and Depression Scale-Anxiety; HADS-D: Hospital Anxiety and Depression Scale-Depression; MFHW: Marburg Questionnaire on Habitual Well-being; PSQI: Pittsburgh Sleep Quality Index.

**Table 3:** Pain pressure thresholds case/control group T0.

		N	MW	SD	SEM	*p*
PPT Facet joints [kPa]	Case	60	222.08	119.496	15.427	0.047
	Control	29	276.70	120.326	22.344	
PPT Deltoid muscle [kPa]	Case	60	230.66	96.704	12.484	0.044
	Control	29	278.52	116.429	21.620	
PPT Tibialis anterior muscle [kPa]	Case	60	270.70	106.779	13.785	0.046
	Control	29	318.87	102.353	19.006	

N: number; M: mean; SD: standard deviation; SEM: standard error of the mean; P: *p*-value; PPT: pain pressure threshold.

**Table 4:** Influence of multimodal pain therapy on pressure pain thresholds.

			MW	SD	SEM	*p*
PPT Facet joints [kPa]	Case	T0	222.08	119.496	15.427	0.005
		T1	256.33	116.098	14.988	
	Control	T0	276.70	120.326	22.344	0.006
		T1	321.07	107.573	19.976	
PPT Deltoid muscle [kPa]	Case	T0	230.66	96.703	12.484	0.025
		T1	212.39	91.527	11.816	
	Control	T0	278.52	116.429	21.620	0.123
		T1	255.06	107.512	19.965	
PPT Tibialis anterior muscle [kPa]	Case	T0	270.70	106.779	13.785	<0.001
		T1	237.27	92.713	11.969	
	Control	T0	318.87	102.353	19.006	0.133
		T1	297.10	103.604	19.239	

M: mean; SD: standard deviation; SEM: standard error of the mean; P: *p*-value; PPT: pain pressure threshold.

**Table 5:** Correlations of demographics, questionnaire scores and PPTs.

		Age	Gender ♂	BMI	HADS-D	HADS-A	PI	v. Korff	MFHW	PSQI
Age	r	1000	0.106	0.001	−0.035	−0.256 *	−0.191	−0.059	0.130	−0.150
	P		0.319	0.996	0.742	0.015	0.071	0.580	0.231	0.163
	n	90	90	84	90	90	90	90	87	88
Gender ♂	r	0.106	1000	0.071	0.164	0.111	−0.012	−0.013	0.080	−0.213
	P	0.319		0.521	0.123	0.298	0.908	0.905	0.464	0.046
	n	90	90	84	90	90	90	90	87	88
BMI	r	0.001	0.071	1000	0.112	−0.058	−0.070	0.043	0.024	−0.100
	P	0.996	0.521		0.311	0.599	0.526	0.695	0.832	0.364
	n	84	84	84	84	84	84	84	81	84
ÖMPSQ	r	0.109	0.035	0.137	0.556 **	0.413 **	0.437 **	0.419 **	0.445 **	0.241
	P	0.306	0.741	0.215	0.000	0.000	0.000	0.000	0.000	0.024
	n	90	90	84	90	90	90	90	87	88
PPT Facet Joints	r	0.380 **	0.255 *	−0.165	−0.161	−0.181	−0.265 *	−0.139	0.194	−0.413 **
	P	0.000	0.015	0.134	0.130	0.088	0.011	0.193	0.071	0.000
	n	90	90	84	90	90	90	90	87	88
PPT Deltoid muscle	r	0.031	0.395 **	−0.062	−0.099	−0.054	−0.063	−0.010	0.151	−0.417 **
	P	0.773	0.000	0.576	0.355	0.617	0.560	0.926	0.166	0.000
	n	89	89	83	89	89	89	89	86	87
PPT Tibialis anterior muscle	r	0.058	0.339 **	−0.309 **	−0.168	−0.116	−0.045	−0.017	0.089	−0.368 **
	P	0.584	0.001	0.004	0.114	0.275	0.673	0.877	0.415	0.000
	n	90	90	84	90	90	90	90	87	88

Gender ♂: male; BMI: body mass index; HADS-D: Hospital Anxiety and Depression Scale-Depression; HADS-A: Hospital Anxiety and Depression Scale-Anxiety; PI: pain intensity; v. Korff: grade of severity; MFHW: Marburg Questionnaire on Habitual Well-being; PSQI: Pittsburgh Sleep Quality Index, r: Pearson; P: *p*-value; n: number; ÖMPSQ: Örebro Musculoskeletal Pain Screening Questionnaire; PPT: pain pressure threshold. * Correlation is significant at level 0.05 (two-sided), ** Correlation is significant at level 0.01 (two-sided), both shaded gray. Metric correlations were represented by Pearson’s correlation coefficient. The correlations of the metric variables with gender are represented by the coefficient Eta.

## Data Availability

The data presented in this study are available on reasonable request from the corresponding author.
